# LIMPRINT in Specialist Lymphedema Services in United Kingdom, France, Italy, and Turkey

**DOI:** 10.1089/lrb.2019.0021

**Published:** 2019-04-22

**Authors:** Vaughan Keeley, Peter Franks, Isabelle Quéré, Gregoire Mercier, Sandro Michelini, Marina Cestari, Pinar Borman, Andrew Hughes, Kath Clark, Jill Lisle, Margaret Benson, Susan Nøerregaard, Tonny Karlsmark, Susie Murray, Christine Moffatt

**Affiliations:** ^1^ Lymphoedema Service, Royal Derby Hospital, Derby, United Kingdom.; ^2^ School of Health Sciences and Medicine, University of Nottingham, Nottingham, United Kingdom.; ^3^ Centre for Research & Implementation of Clinical Practice, London, United Kingdom.; ^4^ Montpellier Medecine Vasculaire, EA2992, Universite Montpellier I, CHU Saint Eloi, Montpellier, France.; ^5^ Dipartimento di Medicina Fisica e Riabilitativa, Ospedale San Giovanni Battista—ACISMOM, Roma, Italy.; ^6^ Study Centre Pianeta Linfedema, Terni, Italy.; ^7^ Department of Physical Medicine and Rehabilitation (PMR), University of Hacettepe Faculty of Medicine, Ankara, Turkey.; ^8^ Hacettepe University Lymphedema Research and Practice Center, Ankara, Turkey.; ^9^ St. Oswalds Hospice, Newcastle Upon Tyne, United Kingdom.; ^10^ LOROS, Leicester, United Kingdom.; ^11^ Copenhagen Wound Healing and Lymphoedema Centre, Bispebjerg Hospital, Kobehavn, Denmark.; ^12^ School of Social Sciences, Nottingham Trent University, Nottingham, United Kingdom.

**Keywords:** specialist lymphedema services, LIMPRINT, primary lymphedema, secondary lymphedema

## Abstract

***Background:*** There is no standardized international model for specialist lymphedema services, which covers the types of lymphedema treated and the treatments provided. The aim of this study was to provide a profile of patients attending specialist lymphedema services in different countries to explore similarities and differences.

***Methods and Results:*** The LIMPRINT core tool was used in specialist lymphedema services in the United Kingdom, France, Italy, and Turkey. Services in Turkey saw a slightly younger age group, with a higher proportion of female patients reflecting a particular focus on breast cancer-related lymphedema. There were higher levels of obesity and restricted mobility in patients in the United Kingdom compared with other countries. Italy and France saw the highest percentage of patients with primary lymphedema. Diabetes was a common comorbidity in the United Kingdom and Turkey. The United Kingdom saw the largest number of patients with lower limb lymphedema.

***Conclusions:*** The results show a wide range of complexity of patients treated in specialist lymphedema services. Some of the differences between countries may reflect different stages in the evolution of specialist lymphedema services, rather than a true difference in prevalence, with those with “younger” services treating a high proportion of patients with cancer and those with more established services treating a wider range of different types of lymphedema, including more elderly people with multiple comorbidities.

## Introduction

In many countries, specialist lymphedema services have been developed to treat patients with chronic edema. However, there is no standardized service model, which covers the types of chronic edema treated, the diagnostic tests available, the range of treatments offered, and the expertise and background of specialist professionals, who make up the multidisciplinary team.

In a number of services, the types of chronic edema treated have evolved over the years. For example, in the United Kingdom, specialist services in the past were largely focused on treating patients with cancer-related lymphedema. Some were based in specialist palliative care services, whereas others developed in specialist medical areas such as dermatology. However, with time, the types of patients treated by many of the services have changed to include primary lymphedema and secondary lymphedema, with a wide range of non-cancer causes including immobility and neurological conditions. A number of services now treat both adults and children. As a result of this, the proportion of patients with cancer-related lymphedema has reduced in these services, as the total number of patients seen has increased.

To facilitate a better understanding of these issues and to enable governments to provide appropriate specialist services for people with chronic edema, a profile of patients attending specialist lymphedema services in different countries has been obtained by using the LIMPRINT methodology. With the wealth of data obtained from such an approach, it is also possible to begin to look at specific features in elements of the population served, for example, the prevalence of cellulitis in people with primary lymphedema compared with those with secondary chronic edema.

## Materials and Methods

Data using the LIMPRINT core tool were obtained from clinical records of all patients currently attending nine specific specialist lymphedema services in four different countries—the United Kingdom, France, Italy, and Turkey. All services checked the accuracy of “current” patients by removing those who had died or had been discharged from the total.

All patients had already been assessed by a specialist team to define the correct classification of chronic edema. Data queries were checked with service leads and lymphedema therapists. As in other LIMPRINT studies, the term “secondary lymphedema” is used to cover all types of chronic edema other than primary lymphedema.

The data were analyzed to provide a description of the total sample, comparisons of patient profiles between countries and between types of chronic edema. Comparisons between countries and types of chronic edema were analyzed by Chi-squared tests or Fisher's exact test, where appropriate.

## Results

### Combined data from all services

#### Total number of patients

A total of 8140 clinical records were assessed in 9 services in 4 countries. Table 1 shows the distribution of patients across the services and countries.

**Table 1.** Distribution of Patients by Service and Country

*Country*	*Service 1*	*Service 2*	*Service 3*	*Total*
United Kingdom	3223	1636	801	5660
France	303	282	—	585
Italy	729	690	—	1419
Turkey	402	74	—	476

#### Distribution of patients by age and gender

**FIG. 1.**
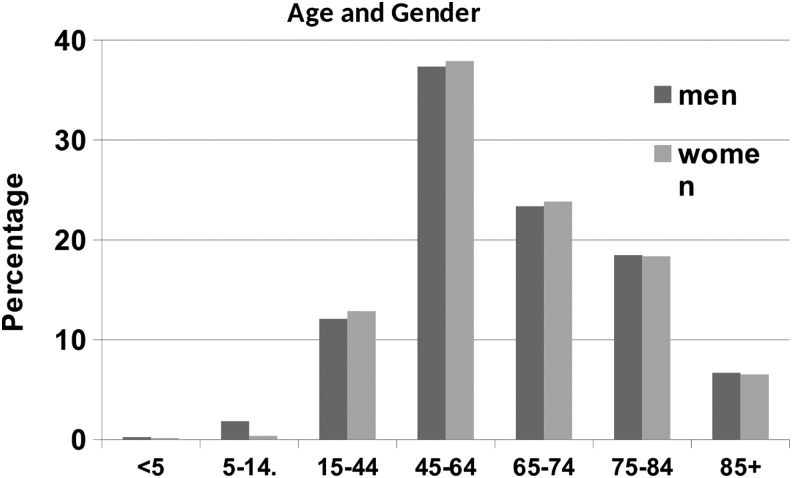
Age and gender.

The distribution of patients by age and gender is shown in Figure 1. The distribution of men and women was similar for all age groups; however, there was a high percentage (79%) of women in the sample. Only a very small percentage of patients were less than the age of 15 years (*n* = 68, 0.84%).

#### Body mass index

In total, 34.5% of patients were obese (body mass index [BMI] = 30–39.9) and 18.4% were morbidly obese (BMI >40). The BMI of 18.5–29.9 includes normal weight (18.5–24.9) and overweight (25–29.9). Only 1.1% of patients were considered “underweight” (BMI <18.5).

#### Mobility

**FIG. 2.**
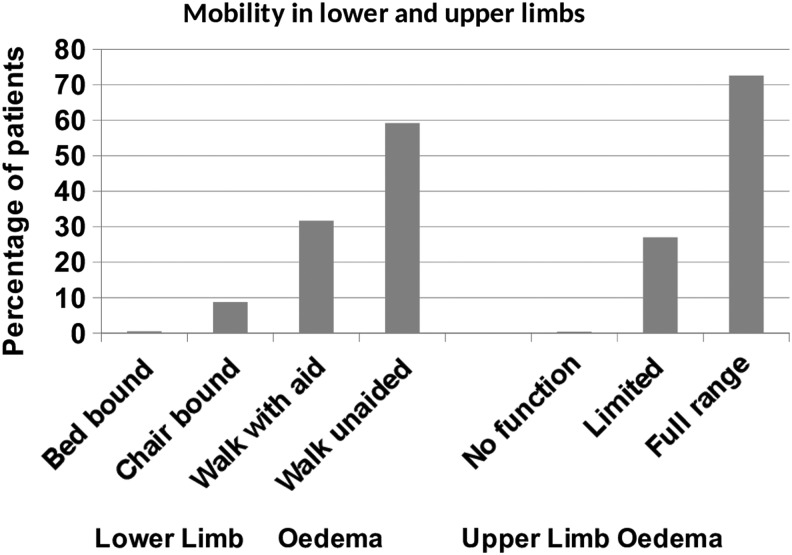
Mobility in lower and upper limbs.

Lower limb mobility was restricted (i.e., walked with aid, chair bound, or bed-bound) in 40.8% of those with leg lymphedema, and upper limb mobility was restricted in 27.4% of those with arm lymphedema (Fig. 2).

#### Cause of chronic edema and comorbidities

A total of 17.5% of patients had primary lymphedema whereas 82.5% had secondary lymphedema. Of those with secondary disease, cancer was the cause in 37.1%. Of the cancer patients, the treatment was the cause of swelling in 95.2%, with metastatic disease accounting for 6.3%. In 60 patients, both cancer treatment and metastatic disease were considered to cause the swelling.

Table 2 shows the factors considered important in the cause of chronic secondary edema. An individual patient may have more than one contributory cause. Comorbidities were common, with diabetes being present in 14.3%, heart disease in 10.3%, and neurological disorders in 5.9% of the total patient group.

**Table 2.** Factors Contributing to Secondary Edema

*Factor*	*%*
Cancer treatment	34.8
Metastatic cancer	2.3
Venous disease	19.6
Immobility	16.3
Obesity	14.3
Other	25.9

#### Duration of swelling

The duration of swelling at the time of assessment is shown in Table 3. More than half (55%) of the patients had had their edema for more than 5 years.

**Table 3.** Duration of Swelling

	*%*
<6 Months	4.8
6–12 Months	6.3
1–2 Years	11.4
2–5 Years	22.6
5–10 Years	23.5
>10 Years	31.5

#### History of cellulitis

In all, 2821 (34.7%) of patients had had at least 1 episode of cellulitis in the past and 1091 (13.4%) had had at least 1 episode within the past year. Of those who had had cellulitis in the past year, 686 (62.9%) had had 1 episode, 253 (23.2%) had had 2 episodes, and 152 (13.9%) had had 3 episodes or more within the past year.

In the previous year, 255 (23.4%) of the patients with an infection were admitted to hospital to manage their cellulitis, of whom 197 (77.2%) were admitted once, 33 (12.9%) twice, and 15 (5.9%) more than twice.

#### Site of swelling and presence of wound

Of the 8140 patients, 7842 (96.3%) had an area of swelling recorded. Of these, 5711 (72.8%) of patients had lower limb edema, 2184 (27.9%) upper limb edema, and 1042 (13.3%) midline edema. Some patients had more than one site of edema.

In total, 543 out of 7842 (6.9%) of patients had non-surgical-associated wounds, though the prevalence of a wound varied greatly between countries (1.7%–7.82%).

### Comparison by country

#### Age profile by country

**FIG. 3.**
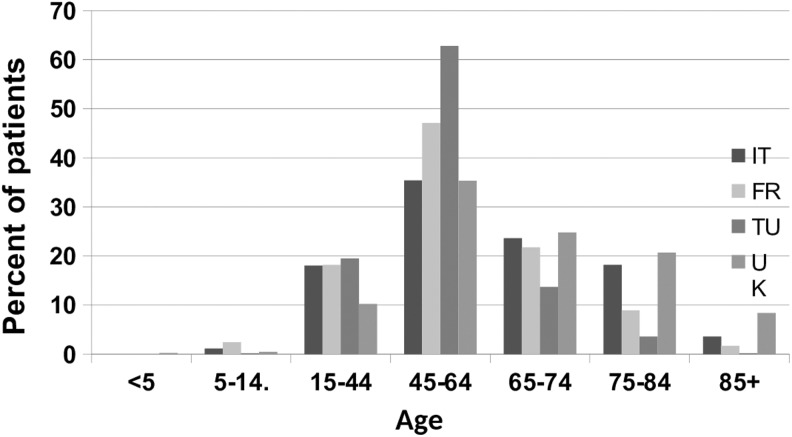
Age profile given by country.

The age profile of patients seen by lymphedema services in each country is compared in Figure 3. The Turkish services saw patients with a slightly younger age group than the other countries, whereas the UK services saw a larger number of older patients, particularly those more than 75 years of age.

#### Gender profile by country

**FIG. 4.**
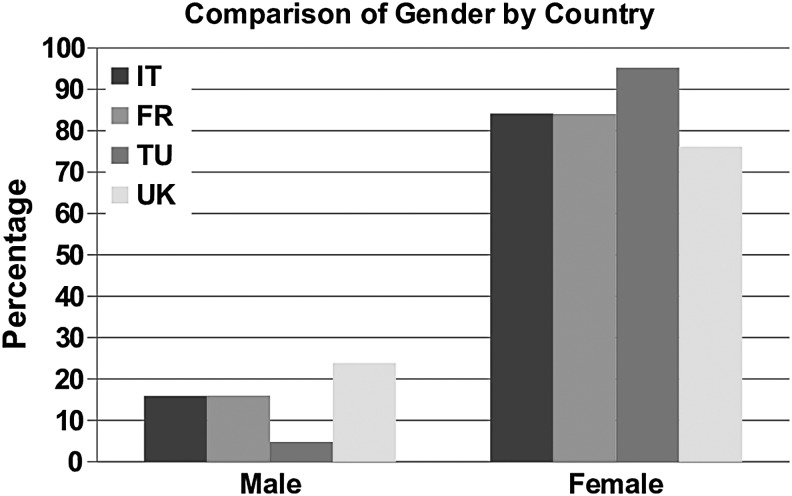
Comparison of gender by country.

Of all the patients seen by Turkish specialist services, 95.2% were female compared with 76.1% in the United Kingdom (Fig. 4).

#### Levels of obesity by country

**FIG. 5.**
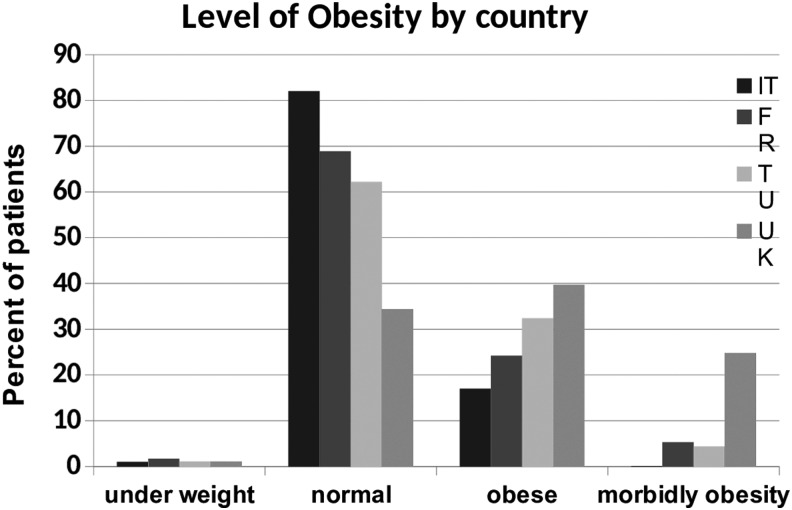
Level of obesity.

There were higher levels of both obese (39.7%) and morbidly obese patients (24.9%) in the United Kingdom compared with other countries. The lowest levels of obesity were found in Italy, with obese patients representing 17.0% of the total and morbidly obese representing 0.1% (Fig. 5).

#### Mobility

**FIG. 6.**
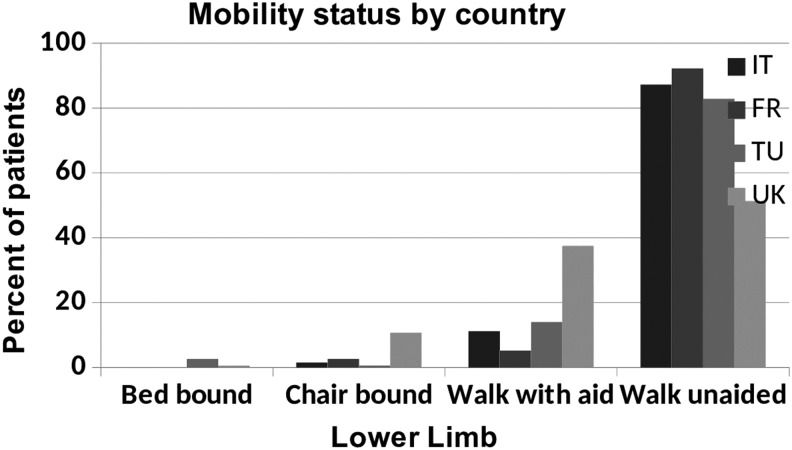
Mobility status by country.

More patients had restricted mobility in the United Kingdom compared with the other countries, with only 51% of those with lower limb lymphedema being able to walk unaided compared with more than 80% in the other countries (Fig. 6).

#### Causes of chronic edema

**FIG. 7.**
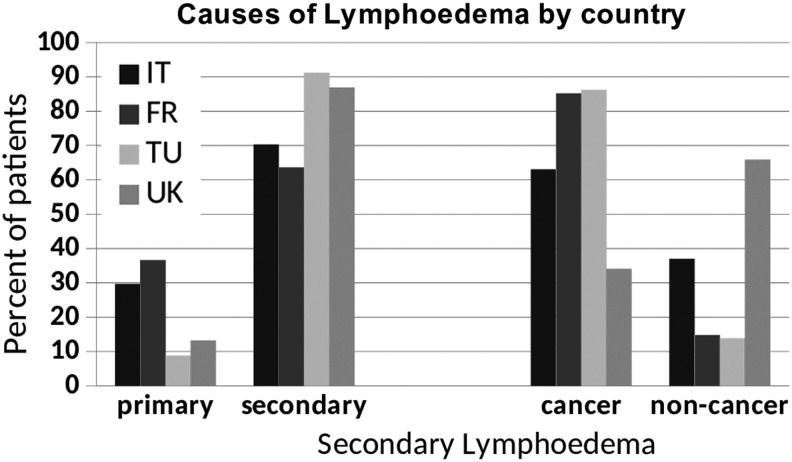
Causes of lymphedema.

Italy and France had higher numbers of patients with primary lymphedema (29.7% and 36.6%) compared with just 8.8% in Turkey. Of the secondary group, cancer-related lymphedema represented around 80% of those seen in France and Turkey but around 34% of those seen in the United Kingdom. Conversely, non-cancer-related secondary lymphedema represented about 66% of those seen in the UK specialist services compared with just 15% of those seen in France and Turkey (*p* < 0.001) (Fig. 7).

#### Comorbidities

**FIG. 8.**
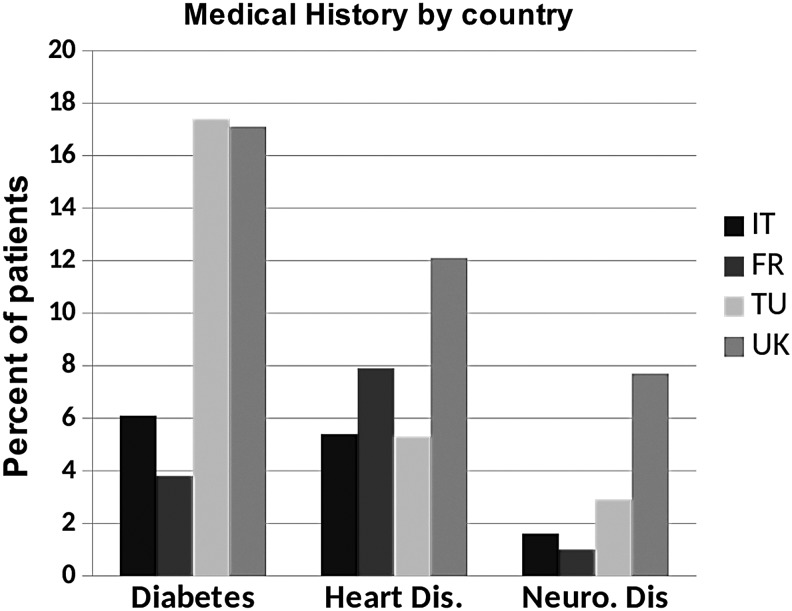
Medical history by country.

Diabetes was present more frequently in patients seen in the United Kingdom and Turkey (17.1 and 17.4%, respectively) compared with 3.8% in France and 6.1% in Turkey. Heart disease was higher in the UK group (12.1%) compared with all other countries (all <8%). Neurological diseases were also more common in patients seen by UK services (7.7%) compared with the other countries (all <3%; *p* < 0.001) (Fig. 8).

#### Duration of chronic edema

**FIG. 9.**
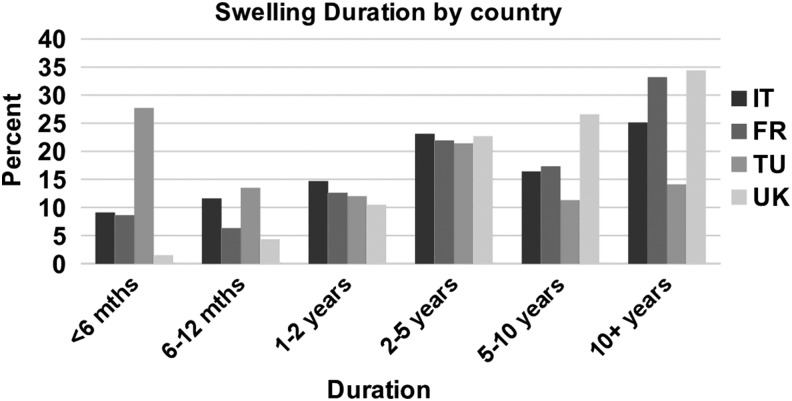
Swelling duration by country.

Italy and Turkey saw patients with a shorter duration of chronic edema than France and the United Kingdom. In France and the United Kingdom, more than 30% of patients had had chronic edema for more than 10 years (Fig. 9).

#### Site of swelling and presence of wounds

**FIG. 10.**
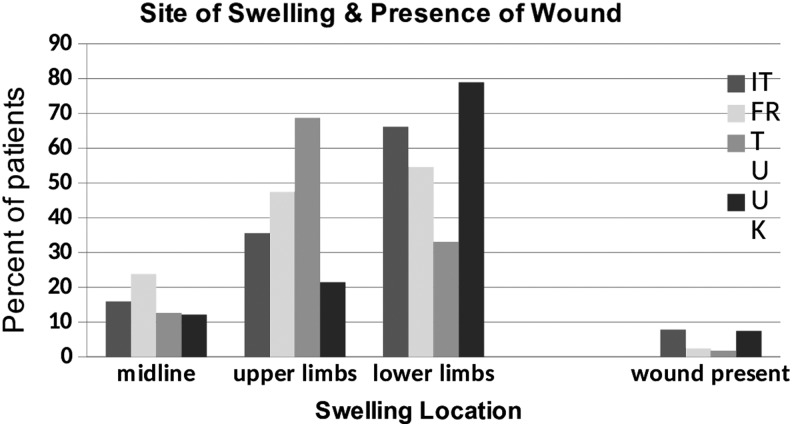
Site of swelling and presence of a wound by country.

Turkey saw the greatest number of patients with upper limb lymphedema (68.7%), whereas the United Kingdom saw the greatest number with lower limb lymphedema (78.9%) (Fig. 10).

Non-surgical wounds were present more frequently in Italy (7.82%) and the United Kingdom (7.37%), with the other countries having fewer than 3% with wounds.

### Comparison of some of the features of primary and secondary lymphedema

#### Episodes of cellulitis

A history of cellulitis was reported in 32.2% of those with primary lymphedema compared with 35.1% of those with secondary lymphedema.

#### The presence of wounds

Wounds were seen in 7.66% of those with secondary lymphedema compared with only 3.22% of those with primary lymphedema across all services and countries (*p* < 0.001).

#### Presence of obesity

Overall, 32.8% of those with primary lymphedema and 34.8% of those with secondary lymphedema were obese whereas 11.2% of people with primary lymphedema and 19.6% of those with secondary lymphedema were morbidly obese (*p* < 0.001) across all categories.

#### Presence of diabetes

Of those with secondary lymphedema, 15.8% had coexistent diabetes compared with 7.0% of those with primary lymphedema (*p* < 0. 001).

## Discussion

These LIMPRINT data have provided a wealth of information about patients who are currently seen in a number of specialist services across Europe. It was already known that there was no standard definition of what constituted a specialist service, for example, staffing, diagnostics, and treatment provision, and which type of patients are seen by such a service.

The collected data from all the services give an overall profile of patients seen and because of the large numbers, they can give some information about other features such as whether there are different characteristics found in those with primary lymphedema compared with those with secondary chronic edema.

### Combined data from all services

This confirms the wide age range of patients seen by specialist lymphedema services and the relatively small number of children seen. Some specialist services do not see children at all.

Not surprisingly, most patients seen were female. This finding is consistent with other published prevalence data.^1^

Fifty-four percent of patients were obese or morbidly obese. This confirms the significant impact of obesity on chronic edema. Managing chronic edema in this group of patients can be challenging in that response to conventional decongestive lymphedema therapy is often disappointing. These data are also important to consider when planning service provision.

Lower limb mobility was restricted in more than 1 out of 3 of those with lower limb lymphedema. Immobility can cause lower limb lymphedema but conversely chronic edema impairs mobility. The role of exercise and physiotherapy input to specialist services is, therefore, important in managing these patients effectively.

Of the total, 16% of patients seen in specialist services had primary lymphedema. This may be a disproportionately high number compared with the prevalence in the wider population as a number of specialist services specialize in managing patients with primary lymphedema.

Cancer and its treatment were the cause of edema in 37% of patients. However, cancer is often considered the major cause of lymphedema by health care providers and these data confirm that, although an important problem, the majority of patients with chronic edema do not have cancer. Venous disease, immobility, and obesity are significant factors contributing to the cause of chronic swelling. Diabetes, heart disease, and neurological disorders were common comorbidities.

This snapshot profile has also confirmed that people attending specialist services have had chronic edema for a varied period. More than half have had swelling for more than 5 years before referral.

Cellulitis is a well-known complication of chronic edema, and more than one third of patients in this study had had at least one episode of cellulitis in the past.

The majority of patients (70%) had lower limb edema. This mirrors the results of previous studies.^1^ In addition, 6.9% of all patients had wounds. This association has been previously demonstrated.^1^

### Comparison by country

There were a number of differences that emerged when the data were analyzed by country.

The Turkish centers saw people of a slightly younger age range than other countries, whereas the UK services saw a large number of older patients, particularly those older than 75 years. More women were seen by the Turkish services (95%). Of those with secondary lymphedema, cancer-related lymphedema represented around 80% those seen in France, Italy, and Turkey compared with around 35% in the United Kingdom. A higher percentage of patients seen in Italy and France had primary lymphedema (30%) compared within Turkey and the United Kingdom (10%). More people in the United Kingdom had restricted mobility than those in other countries. In addition, diabetes, heart disease, and neurological disease were more common in patients seen in the United Kingdom. Italy and Turkey saw patients with a shorter duration of chronic edema than France and the United Kingdom. The Turkish services saw the greatest number of patients with upper limb lymphedema (69%), whereas UK services saw the greatest number of patients with lower limb lymphedema (79%).

Of the patients seen in Italy, 7.82% had wounds with a similar proportion in the United Kingdom. The remaining countries had fewer than 3%.

The Turkish centers are largely focused on seeing women with breast cancer treatment-related lymphedema. Conversely, the UK services see large numbers of patients with chronic secondary edema related to a wide range of other conditions, which occur in more elderly populations. Diabetes seemed to be disproportionately common in the Turkish lymphedema services, but this is consistent with a higher prevalence of diabetes in Turkey's general population than in the other countries represented here. According to a WHO report, the prevalence of diabetes in Turkey is 13.2% compared with 7.7% in the United Kingdom, 8.5% in Italy, and 8.0% in France.^2^

The United Kingdom had the highest proportion of people with obesity and morbid obesity. This fits with OECD figures, which show a prevalence of obesity in 2017 as 10% in Italy, 15% in France, 22% in Turkey, and 27% in the United Kingdom.^3^

The differences in patients seen between countries may, in part, not only reflect the different prevalence of other conditions such as obesity and diabetes but also probably reflect the types of patients referred to and seen by the different specialist services. Historically, in the United Kingdom, many developing lymphedema services in the 1980s and 1990s focused on treating people with cancer.

However, subsequently, UK services began to see more and more patients with non-cancer-related chronic edema and this is reflected in the UK figures seen in this study. This probably reflects more closely the range of different types of chronic edema present in community populations. Countries such as Turkey may be undergoing a similar evolutionary process, as it will be increasingly recognized that those with cancer-related lymphedema represent only a minority of people who have chronic edema. However, it is possible that patients with non-cancer-related edema may be seen by other services not taking part in this study.

### Comparison of features of primary and secondary lymphedema

This large dataset has enabled an analysis that can compare problems associated with primary and secondary lymphedema. A history of cellulitis was seen in around 35% of both those with primary lymphedema and those with secondary lymphedema, suggesting that there is little difference between the two types, and that patients with both primary and secondary lymphedema should be advised about the risk of cellulitis and how to manage it. Wounds were seen more frequently in those with secondary lymphedema. This is consistent with previous data.^1^

Interestingly, the percentage of people with primary and secondary lymphedema who were obese was similar, although morbid obesity was present in 19.6% of those with secondary lymphedema compared with 11.2% of those with primary lymphedema. This stresses the importance of obesity not only as a cause of chronic edema but also as a contributory factor in making lymphedema worse.^4^ Diabetes was more common in those with secondary lymphedema than those with primary lymphedema. This could, of course, be related to the increased risk of type II diabetes in obesity.

Although the methods adopted for the LIMPRINT study and the training of those carrying out the work should facilitate a consistent approach across countries, it is possible that there could be some inconsistencies in the diagnosis of primary lymphedema. Primary lymphedema is currently considered a genetic condition and is often diagnosed by using the St George's algorithm.^5^ In the past, however, it was considered a diagnosis of exclusion and, therefore, if no secondary cause of chronic edema was found, the condition was labeled as “primary.” However, it is not clear whether these factors may have influenced the diagnosis of primary lymphedema here.

## Conclusions

The data reported here show the wide range of complexity of patients seen in specialist lymphedema services. This needs to be reflected in the skills of the multidisciplinary team providing the service. The data could, therefore, be useful to inform the development of a service specification for specialist chronic edema services.

Some of the differences between countries may reflect different stages in the evolution of specialist lymphedema services rather than big differences in the prevalence of different types of chronic edema in the communities served by the services. Population-based studies would help give an answer to this but are extremely difficult and costly to carry out.
